# Curricular integration of virtual patients: a unifying perspective of medical teachers and students

**DOI:** 10.1186/s12909-019-1849-7

**Published:** 2019-11-09

**Authors:** Eleni Dafli, Ioannis Fountoukidis, Chariklia Hatzisevastou-Loukidou, Panagiotis D Bamidis

**Affiliations:** 1 School of Medicine, Faculty of Health Sciences, Aristotle University of Thessaloniki, PO Box 376, 54124 Thessaloniki, Greece; 2 School of Economics, Faculty of Economic and Political Sciences, Aristotle University of Thessaloniki, 54124 Thessaloniki, Greece

**Keywords:** Virtual patients, Medical curriculum, Medical education

## Abstract

**Background:**

Virtual Patients (VPs) may improve cognitive and behavioral skills better than traditional methods do. The aim of this paper was to investigate challenges faced by teachers and students in order to effectively implement VPs across undergraduate and postgraduate curricula. In addition, differences in student and teacher perceptions that could impact curricular integration of VPs were explored.

**Methods:**

A two-phase descriptive study was performed: 1) evaluation of the VP design process and curricular integration, conducted upon academic medical teachers; 2) evaluation of learning and clinical reasoning experiences with VPs, from the students’ perspective.

**Results:**

The results of this study document high acceptance of VPs by both medical teachers and students (*n* = 252).VPs seem to fulfill most needs as set by course directors, while they satisfy student needs and create perceptions of improved knowledge and clinical skills reasoning.

**Conclusions:**

Medical educators have encountered educational challenges upon transforming the curriculum. To develop VPs, academic institutions have to pay equal attention to the needs of potential adopters and VP authors. Strategic development and use of VPs may motivate more widespread integration of VPs and lead to a high quality medical education system.

## Background

Even though the rapid increase in medical information and the expectations for quality health care during the last decades have enhanced the complexity of medical decision-making, there has been a reduction of time for education [1] and strong concerns about the patients use as teaching subjects [2]. Safer and more effective means are therefore needed to support the development of clinical reasoning skills required in medical education. Moreover, medical training has now become more student-centred [3] and more emphasis is given to active learning and clinical skills reasoning rather than passive knowledge acquisition and fruitless recall of information [4–6].

The rapid development of new reliable information technologies enabled the creation of contemporary learning activities that could not be previously achieved. These can now enhance effectively medical education and training [7]. Under this perspective, Virtual Patients (VPs), often defined as “specific types of computer-based programs that simulates real-life clinical scenarios where learners emulate the roles of health care providers to obtain a history, conduct a physical exam, and make diagnostic and therapeutic decisions” [8], have been increasingly used as educational resources in many medical educational institutions [9]. The main features of VP systems are that they provide practice in a safe and no patient risk environment and they can be repeatedly used by any clinical skills learner without regard to time and place [10].

These opportunities that VPs provide in modern medical education [11], in association with positive evaluation results of various studies that demonstrated that they may improve cognitive and behavioral skills better than traditional methods do [12], led to a wide trend towards VP creation and use among academic institutions [13]. However, the successful integration and effective full adoption of VPs in medical curricula has not been easily implemented in a broad range of medical schools, since it hinges on the extent to which medical educators sufficiently consider curricular issues [14]. Now that rigid educational programs give space to more flexible and adaptable ones, in which the medical teachers’ and students’ feedback at the same time, play an increasingly important role [15], changing the medical curriculum towards a thorough and consistent implementation of VP simulations remains a challenge [16].

The Medical School of Aristotle University of Thessaloniki (AUTH) has made remarkable efforts to improve medical education in the last few years, trying to assimilate new trends and modernize the curriculum. Part of this effort was initiated in the mEducator project (www.meducator.net) [17, 18] only to be materialized then in the ARIADNE project (http://vp.med.auth.gr/ariadne), which resulted in the development of a considerably extensive and open VP repository; the latter aimed at training students in applied knowledge, clinical reasoning and professional attitude, and led to the full incorporation of these newly created educational objects in Problem Based Learning (PBL) sessions in the medical curriculum, both undergraduate and postgraduate. Following the procedure of creation, implementation and incorporation of the VP cases in the curriculum, an effort for a comprehensive evaluation of various parameters of the VP design, use and integration process, from both educators’ and students’ perspective, aimed to add meaningful knowledge considering the full adoption of VPs.

Thus, the aim of this paper was to perform a both quantitative a qualitative research for the VP use and implementation across the medical curriculum. Concerning the quantitative approach, the purposes of the study was a) to measure the propensity of medical teachers and students toward the use of VPs in the medical curriculum and b) to measure the preferences of medical teachers and students concerning the way VPs should be utilized across their studies. At the same time, the qualitative evaluation performed in this study aimed to a) describe advantages and disadvantages in VP integration into the medical curriculum and b) to describe the experiences of both medical educators and students in adopting VP resources in their learning and training across the medical curriculum. This bilateral perspective could help in shaping new strategies to effectively integrate this unique type of educational resources in medical education, while addressing important questions on the optimal design and use of VPs. A well-designed curriculum integration of VPs will have the potential to generate medical students with professional knowledge, experiential learning and critical thinking.

## Methods

### Context of the study

The ARIADNE project team consisted of 34medical teachers (lecturers, professors and scientific associates of the Medical faculty), in collaboration with 5 members of the mEducator project and the Medical Education Informatics (medphys.med.auth.gr/mei) research team who, apart from the technical support of the system, undertook the responsibility of “training the trainers” or teacher training with respect to technical knowledge and skills required for VP authoring. The outcome of this effort was the creation of a repository of the first 35 VP cases in the Greek academia as well as the local Medical School. These VP cases were implemented through OpenLabyrinth, an open source on line activity modeling system that allows the design and implementation of interactive “game informed” educational activities [19]. These cases were intended to be built around enquiry-based approaches to learning, something that was supported by the VP authoring system. Thus, a variety of features were available to the authors, such as, multiple nodes and links, avatars, media files, rules, info buttons, counters and skins, all these provided through a user friendly interface and a visual editor. Finally, depending on how familiar each member of the faculty was with modeling systems, each VP was created after 1 to 2 months’ effort. Open exploratory access to this VP repository was initially given to undergraduate medical students and subsequently, after positive preliminary feedback, the VP cases were formally incorporated in the medical curriculum as part of pilot small group of PBL sessions. At the same time, the repository was enriched by adding new cases, in an effort to include VPs applied to a wider variety of medical specialties. Today, the database counts some 59 VP cases.

Evaluating how medical teachers and students use VPs is crucial for the effective development of these educational resources [20]. To explore medical students’ and teachers’ perception of VPs’ integration in medical curriculum, a descriptive study was designed and performed [21], as an attempt to explore and explain, while providing additional information, about VP design and implementation from both sides, medical students’ and teachers’ as well. The evaluation performed at the ARIADNE project included two parts: 1) the evaluation of the VP design process and curricular integration, conducted upon the medical teachers participating in the design and implementation of VPs and 2) the evaluation of the learning and clinical reasoning experiences with VPs, conducted upon students, using the 59 virtual patients that currently exist in the repository.

### Approach and researcher characteristics

This evaluation aimed to include both quantitative and qualitative data, so as to form a mixed method approach that will provide answers in the specific research questions previously mentioned in the introduction. Specifically, as far as the quantitative approach is concerned, an effort for recording the medical teachers’ and students’ position and tension for the adoption of VPs was made and, at the same time, their preferences for the VPs’ best utilization as learning activities were registered. On the other hand, for the qualitative approach, content analysis was used as a method. Open-ended questions led to a wide variety of transcripts which had to be analyzed in a qualitative oriented way. Categories were not established before beginning the analysis but were allowed to emerge during analysis. Coding the data was the process of organizing the results in a comprehensible way. During the analysis, more coders were used to ensure the trustworthiness of the analysis. Medical teachers were asked to identify the advantages and the weaknesses of VP integration and describe their experiences and preferences with VPs. At the same time, medical students were asked to evaluate their learning and clinical reasoning experiences with VPs, so as the pros and cons of VPs to be highlighted. Moreover, both medical teachers and students were asked to provide us with their preliminary experiences for the expected VPs’ integration across the medical curriculum.

Concerning the main researchers, ED is a medical doctor with experience in medical educational simulations and IF is a specialist in healthcare management and informatics. Both ED and IF participated in the data collection and analysis and had no personal relationships with the medical teachers and students that took part in the study.

### Study population

In total, 252 participants, both medical teachers and students, participated in the evaluation procedure of VPs’ curriculum integration.

**Fig. 1:**
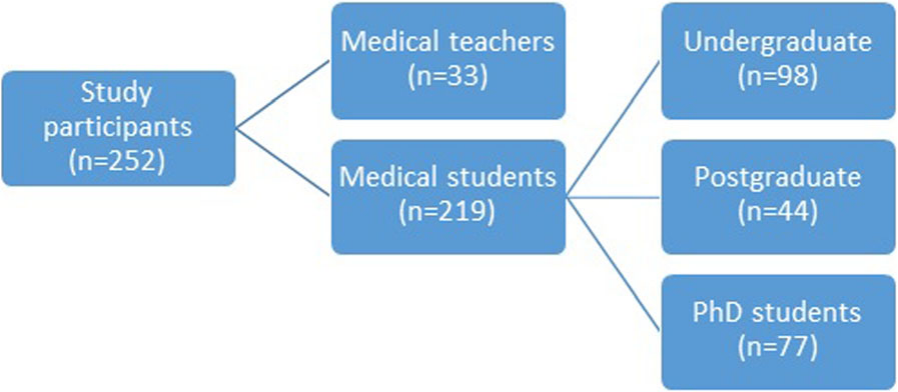
In total, 252 evaluators, 33 medical teachers, in the first phase, and 219 medical students, in the second phase, participated in the evaluation. The second phase included 98 undergraduate medical students, 44 postgraduate medical students and 77 PhD students

In the first phase of evaluation, all medical teachers attending the ARIADNE project participated in the study (*n* = 33). In the second phase, 219 participants have arisen from a random selection of medical students (undergraduate, postgraduate and PhD students) (Fig. 1).

### Instruments and data collection

The study included the collection of quantitative and qualitative data, using two on line available questionnaires, for the medical teachers’ and students’ evaluation, accordingly. Data were collected between September 2017 and December 2017 using face-to-face groups.

For the medical teachers’ evaluation of the VP design process and curricular integration, an 11-item, web-based questionnaire, including Likert-type, open-ended, dichotomous and multiple choice questions, was developed and utilized, aiming to assess the design, implementation and adoption of VP cases (Table 1). Firstly, medical education experts of the School of Medicine were asked to read and evaluate whether the questions effectively capture the topic under investigation. The next step was to pilot test the survey on a subset of the intended population that means medical teachers.

**Table 1:** The medical teachers’ questionnaire for the VPs creation, implementation and adoption

Question type	Question	Answer
Dichotomous	Are educational activities based on virtual patients more interesting than traditional ones?	Yes/No
Dichotomous	Did the design process of a virtual patient increase your research interest for the clinical condition related to the case?	Yes/No
Open-ended	What are the advantages of the virtual patient design and implementation process and how do they enhance learning?	Free text
Open-ended	What are the weaknesses of the virtual patient design and implementation (or which parts can be improved to contribute more effectively to the educational process)?	Free text
Open-ended	Which part of the virtual patient design do you believe is more useful as an educational experience?	Free text
Open-ended	Do you think that the selection of multimedia material contributes, to the educational process? If yes, which way?	Free text
Set of Yes/No Questions	The design process of a virtual patient contributes to:	a. Clinical skills development through proper virtual patient management. b. Clinical skills development through training in decision making. c. Support of the educational experience, since it could be considered equal to training with real patients. d. Increase of gaining knowledge. e. Better memorization of key points. f. Deeper knowledge of the disease.
Likert-scale	What is the difficulty level of the virtual patient design?	1–5 (in ascending order)
Open ended	What do you mostly remember from this activity?	Free text
Dichotomous	Do you consider that the virtual patient design process corresponds to your IT level of knowledge?	Yes/No
Dichotomous	Did the virtual patient design process increase the team spirit?	Yes/No
Dichotomous	Do you think it would be possible to implement this method in other fields?	Yes/No

For the evaluation of students’ learning and clinical reasoning experiences with VPs, the validated eViP evaluation tool kit for use with VPs was used [22]. This student questionnaire consists of 14 questions clustered into the following main categories: a) authenticity of patient encounter and consultation, b) professional approach in the consultation, c) coaching during consultation, d) learning effect, e) overall judgment, f) open-ended questions [23]. Likert scale questions are used in this questionnaire with their statements based on attitudes and cognitive activities. Furthermore, a few questions were added, including those concerning demographic characteristics (sex and age group), the year of study, the title of VPs evaluated and finally, a multiple choice question referring to the integration of VPs into the medical curriculum, including the options: a) as an additional learning activity, b) as an additional tool for practice before the exams, c) as a tool for clinical skills acquisition before real clinical contact and d) as an assessment tool. This instrument was administered to medical students after they encountered a number of VPs.

### Bioethical approval

The Bioethical Committee of the Aristotle University of Thessaloniki approved this study (reference no. 1/22-12-2011).

### Statistical analysis

The on line questionnaire data were entered into a database and then imported into IBM SPSS Version 19.0 (SPSS, Inc., Chicago, IL, USA) for descriptive statistical analysis.

As far as the qualitative data of the study are concerned, the SRQR guidelines were followed, so as the results of the qualitative part of the study to be more easily accessible to readers when critically appraising, applying, and synthesizing study findings.

### Data analysis

Data analysis took place at the same time with data collection. Identifiers were omitted to maintain anonymity and confidentiality. Transcripts were analysed using content analysis. A coding sheet was created following a discussion among the primary investigator and supervisors (PB and CH.). Transcripts were read carefully and a summary of meaning units was generated and reduced into codes. Codes were then transferred to the coding sheet, and similar codes were gathered under sub-categories. A category scheme was then developed to group similar sub-categories together. Finally, themes that connect the various categories were identified.

### Enhancing trustworthiness

Credibility was enhanced by the heterogeneous sample and by performing member check, and using field notes. Dependability was established by having the primary investigator and two experienced researchers review the coding process and agree on the analysis. Confirmability was addressed through constant dialogue among the researchers. Finally, authenticity was ensured by using icebreakers to establish a trusting relationship with the participants and get them to openly discuss their experiences.

## Results

### Medical teachers’ evaluation results

The vast majority of participants (83.3%) approved that educational activities based on VPs are more interesting than traditional ones. Medical teachers in a rate of 60% agreed that the design process of a VP increased their research interest for the clinical condition related to the case. However, some 63% of them responded that the educational experience with VPs cannot be considered equivalent to a real clinical contact, but all of them (100%) agreed that it can contribute to the development of clinical skills through practice in clinical decision making (Table 2). Finally, the majority of participants (some 79%) agreed that their information technology (IT) capacity was adequate for the VP design, while the total of faculty members stated that this educational method would be possible to be implemented in other sectors, too (Table 3). These are the first results of a teachers’ evaluation of VPs’ use and integration and the possibility of measuring more subtle differences in future studies, using more detailed questionnaires, including likert scale questions for example, is a challenging issue.

**Table 2:** Medical teachers’ perceptions for VPs’ design, use and adoption in curriculum

The design process of a virtual patient contributes to:		Table N %
Deeper knowledge of the disease	Yes	48,5%
	No	51,5%
Better memorization of key points.	Yes	51,5%
	No	48,5%
Increase of gaining knowledge	Yes	45,5%
	No	54,5%
Support of the educational experience, since it could be considered equal to training with real patients.	Yes	36,4%
	No	63,6%
Clinical skills development through training in decision making.	Yes	63,6%
	No	36,4%
Clinical skills development through proper virtual patient management.	Yes	60,6%
	No	39,4%

**Table 3:** Medical teachers’ views on the VP design process and implementation

		Table N %
Do you consider that the virtual patient design process corresponds to your IT level of knowledge?	Yes	79,3%
	No	20,7%
Did the virtual patient design process increase the team spirit?	Yes	53,8%
	No	46,2%
Do you think it would be possible to implement this method in other fields?	Yes	100,0%
	No	0,0%

The results of the medical teachers’ questionnaire analysis also contained qualitative data. Medical teachers identified 14 pairs of positive and negative experiences related to virtual patient use. Positive and negative experiences were categorized into two major themes: (1) Educational aspects of VP use and (2) Communication aspects of VP use. Within the two major themes, we identified five subthemes: (1a) Clinical Care of patient (i.e., clinical efficiency), (1b) Learning effect of the VP, (1c) Information Access, (1d) Quality of the educational Resource, (2a) Students engagement and (2b) Student collaboration. Representative medical teacher quotes are provided in Table 4.

**Table 4:** Themes and Representative Quotations of Positive and Negative Medical Teachers’ Experiences with Virtual Patients

Codes	Examples of positive codes	Examples of negative codes
Major Theme (1) Educational aspects of VP use (159)		
Subtheme (1a) Clinical Care of patient (34)		
1.Clinical Efficiency (10)	“It makes the virtual visit go smoother…I take notes and prioritize knowledge… I don’t have to flip through a huge scenario” *n* = 8	“The doctor spends too much time” n = 2
2.Case review (24)	“I can go back and look at important test results” *n* = 23	“There are ECHO reports, but it seems like I don’t find where there are in the scenario” *n* = 1
Subtheme (1b) Learning effect of the VP (55)		
1.Facilitation of knowledge (11)	“The case and virtual trainer was asking me questions to verify and make sure everything is correct” *n* = 10	If they used some graphs or charts, I might say that was more effective” n = 1
2.Medical history (23)	“I had easy access to the vp history at my convenience” *n* = 21	“There are inaccuracies in at each scenario node I go to, they’re not always updating it” *n* = 2
3.Assessment of knowledge (21)	“I think that learning with the virtual patients is important in order to do well in the final exam for this course” *n* = 17	“It does not make you feel secure enough” *n* = 4
Subtheme (1c) Information Access (40)		
1.Accessibility of information (20)	“…with these records online I have easier access” *n* = 19	“Sometimes the computer doesn’t work and I can’t access information.” n = 1
2.Suitable information (20)	“Every node included information as in real life patients” *n* = 19	“Some links to nodes were linked to inappropriate information” n = 1
Subtheme (1d) Quality of educational resource (30)		
1.Authenticity of patient encounter (16)	“While working on this case, it was like I had to make the same decisions a doctor would make” *n* = 15	“I didn’t feel like being a real the doctor caring for a real patient” n = 1
2.Professional approach (14)	“I was gathering the information I needed, to characterize the problem” *n* = 12	“It was difficult to think which findings supported or refuted each diagnosis” n = 2
Major Theme (2) Communication aspects of VP use (59)		
Subtheme (2a) Students engagement (37)		
1.Use of media material (21)	“Videos add realism in the scenario and makes you want to visit quickly the next step” *n* = 20	“Avatars make it seem fake…better with real patients” n = 1
2.Facilitate the user discussion (12)	“You can talk and look at results together in the computer…” n = 8	“Everybody may work alone in front of a PC” n = 4
3.Student questions (4)	“Students might have questions for the teacher that might forget, but going through the VP together may help them to remember their questions” n = 3	“They may read what’s on the screen and forget to say something” n = 1
Subtheme (2b) Student collaboration (22)		
1.Ideas sharing (13)	“Students can discuss on several different decision pathways each scenario includes” *n* = 9	“VPs may not discuss if it used as a self learning process” n = 4
2.Social presence (9)	“You feel like part of a ‘community’ during the corresponding teaching events.” *n* = 6	“Someone may feel insecure to openly share shortcomings” n = 3

Most participants pointed out that VPs provide the opportunity for a more thorough clinical representation of medical educational scenarios, contact with rare clinical cases, distance learning and self-assessment in a controllable learning environment. It was mentioned that “students will have the opportunity to practice in rare cases written from experts of the specific field”. Moreover, it seemed that VPs support PBL, allow for realism and cognitive errors in a safe environment and contribute to critical thinking development. As important part of the realism of the educational environment was considered the sum of wrong choices that as a part of the educational activity offer the chance to students to experiment by following different diagnostic paths and see the consequences of their choices without impact on real patients. Furthermore, it was mentioned that the whole decision tree, including the right choices, as well, contributes to effective learning. They finally pointed out that VPs improve medical education and increase motivation for learning

An important feature that helps in increasing motivation was reported to be the part of media resources, since they add extra value and interactivity to the clinical cases, resulting in more attractive and interesting learning scenarios to trainees. For example it was mentioned “These media files will make the VP very attractive to trainees! They will feel like playing a game with avatars”. Besides, multimedia material was mentioned to add realism to VPs and contribute to a more thorough presentation of clinical cases, leading to better memorization of the diagnostic process. Thus, the optimal use of media adds authenticity and better memorization of clinical conditions.

Another important issue that was highlighted on top of that, however, was that VP design is time and effort consuming and needs, even basic, IT knowledge and support. It was also sporadically mentioned were that VPs as educational objects lack the ability for real contact between instructor and learner and, as far as the design process is concerned, there is a need for a stock of media material for the proper VP creation, thereby indicating the need for VP repurposing and reusing.

### Medical students’ evaluation results

The medical student population included both male (61.20%) and female (38.80%) participants.

**Fig. 2:**
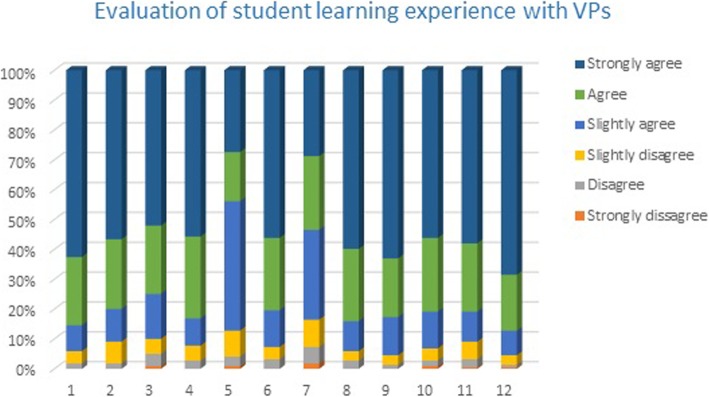
The results from the eViP evaluation tool kit for use with VPs. In all 14 questions the majority of medical students mentioned that agreed that VPs improved their learning experiences focusing on the development of clinical reasoning skills)

In a first phase, results of the overall evaluation from all participants were obtained, regardless of sex, age and year of study (Fig. 2). Most medical students provided positive statements regarding their learning and clinical reasoning experiences with VPs as educational objects and endorsed their curricular integration.

Moreover, the vast majority of participants answered positively to the question of VPs’ integration in the medical curriculum (in a percentage over 98%), something that shows high acceptance of VPs’ incorporation in the curriculum (Table 5).

**Table 5:** Student answers concerning their opinion concerning the integration of VPs in the medical curriculum with regard to sex and year of study

	Year of study	Would you like VPs to be integrated in the medical curriculum?	
		Yes (%)	No (%)
Male	1st-2nd year	100.00	0.00
	3nd- 5^th^year	88.89	11.11
	6^th^year	0.00	0.00
	Pre-degree	100.00	0.00
	Postgraduate	100.00	0.00
	PhD	100.00	0.00
	Total	99.25	0.75
Female	1st-2^nd^year	100.00	0.00
	3nd-5th year	0.00	0.00
	6^th^year	100.00	0.00
	Pre-degree	83.33	16.67
	Postgraduate	93.75	6.25
	PhD	98.04	1.96
	Total	96.47	3.53
Total	1st- 2nd year	100.00	0.00
	3nd- 5^th^year	88.89	11.11
	6^th^year	100.00	0.00
	Pre-degree	85.71	14.29
	Postgraduate	97.73	2.27
	PhD	98.70	1.30
	Total	98.17	1.83

Furthermore, male participants of the first 2 years of study rated their educational experience with VPs with a mean of 5.55 in a scale 1–6, while female students of the same years of study rated their educational experience with a mean of 3.08 in the same scale. Moreover, male and female postgraduate and PhD students rated their educational experience with about the same value that slightly exceeds 5.13. Similarly, males aged under 25 evaluated their experience with a mean of 5.33 and females aged under 25 with a mean of 3.86. These lead to the conclusion that male young students of the pre-clinical years of study show a significant preference in VPs as learning objects, in comparison to their female mates.

Going a step further, it can be easily seen from the results of all participants (Table 4) that students of the pre-clinical years show a high preference in the adoption of VPs as learning activities. That could be explained from the lack of any clinical contact with real patients in their two first years of study and their willingness to have early, even virtual, clinical encounters. At the same time, there is a decrease of interest for VPs’ curricular adoption in the next years of study, something that is reversed again in the last (6th) year of study. This is the time when medical students often desire to expand their clinical experience and practical skills acquisition, since they will soon be active members of the healthcare workforce.

In Table 6, the preferences of medical students concerning the type of use and integration of VPs in the medical curriculum are presented, sorted according sex and year of study. As illustrated, undergraduate students show a strong preference for VPs use as a tool for clinical skills acquisition before real clinical contact, with a 100% rate of acceptance. Additionally, undergraduate students seem to be more willing to adopt VPs as an assessment tool or an additional tool for practice before exams in comparison to postgraduate and PhD students, as expected.

**Table 6:** Student preferences concerning the ways of integration of VPs in the medical curriculum

		Additional learning activity		Additional tool for practice before exams		Tool for clinical skills acquisition before real clinical contact		Assessment tool	
		Yes(%)	No(%)	Yes(%)	No(%)	Yes(%)	No(%)	Yes(%)	No(%)
Male	Undergraduate	18.57	81.43	78.57	21.43	100.00	0.00	87.14	12.86
	Postgraduate	64.29	35.71	46.43	53.57	60.71	39.29	42.86	57.14
	PhD	57.69	42.31	57.69	42.31	53.85	46.15	61.54	38.46
Female	Undergraduate	100.00	0.00	85.71	14.29	100.00	0.00	28.57	71.43
	Postgraduate	31.25	68.75	0.00	100.00	68.75	31.25	0.00	100.00
	PhD	45.10	54.90	52.94	47.06	80.39	19.61	50.98	49.02
Total	Undergraduate	25.97	74.03	79.22	20.78	100.00	0.00	81.82	18.18
	Postgraduate	52.27	47.73	29.55	70.45	63.64	36.36	27.27	72.73
	PhD	49.35	50.65	54.55	45.45	71.43	28.57	54.55	45.45

## Discussion

With the increasingly widespread adoption of VPs, there exist potential pedagogic synergies to allow trainees the opportunity to practice in realistic and safe learning environments. The results of this study are consistent with findings from other studies of VP adoption [24] and even though the number of experimental studies investigating the utility of VPs is limited, these matching results are positive and encouraging [25].

Considering the likelihood that technology will grow increasingly sophisticated and costly, we have to be thoughtful about how limited, available resources are used, and we should strive in unison towards improving the education of our future physicians [26]. The promise of VPs as a method of enhancing the process of clinical skills reasoning has greatly impacted health professions education. At the same time, the opposing forces of increased training expectations and reduced training resources increase the need for future VP developing institutions to consider the role of VPs in the educational armamentarium. Thus, since few academic institutions can afford to develop VP cases, one solution is the development of virtual online communities where resources are shared, repurposed and reused [18, 25].

In the light of our results concerning the VP design, medical teachers seem to be able to develop and edit highly interactive VP cases with comparatively little assistance from computer specialists. Additionally, the process of VP design and development itself provides them the opportunity of further deepening in clinical knowledge and clinical reasoning. The VP creation and implementation process in medical curriculum can also serve as a means for collaboration between medical teachers of the same or different medical specialties and contribute to sharing understanding in health care education.

In terms of the research questions of this study, as far as the propensity of medical teachers and students to use VPs and moreover the implementation and formal integration in the medical curriculum, current VPs seem to fulfill most needs from course directors and teachers from various educational disciplines and medical specialties, while they seem to satisfy student needs and perceptions of improved knowledge and clinical skills reasoning. At the same time, concerning the preferences of VP utilization, the virtual clinical encounters used in this study appear to be particularly well suited for learning and assessment purposes for medical students, who have had limited or no significant clinical contact. Therefore, medical teachers and students, as well, propose a strategic development and use of VPs in underrepresented topics, especially in pre-clinical medical education, since they may motivate a more widespread integration of VPs into medical education [27].

As far as the exploitation of the advantages and disadvantages of VP integration and user experience, the results of this study suggested that the VP learning effect, the contribution to the better clinical care, the better access and the quality of the education resources seem all to support the formal integration of these learning objects to the medical curriculum.

We have to mention that in our case, VP scenarios were not part of the core curriculum, but were actually used as supplementary materials to the formal curriculum. Maybe they would be regarded differently by faculty and learners if they were considered part of the core curriculum.

The creation of additional clinical competency cases for both preclinical and clinical years of study including questions and explanations tailored to the critical-thinking that would allow learners the opportunity to explore the consequences of clinical decisions are highly recommended from both medical teachers and students. In addition, wider development of VPs in other disciplines, such as acute care and surgery, would result in a comprehensive body of cases to complement the entire clinical curriculum. Such a development of the VP instruction model would encompass the progression dimension across a whole medical curriculum, where VPs are present in all curricular semesters in an effort to aid the integration of theory and practice aligned by findings in others studies [4].

This study indicates issues that are deemed highly important for the exploration of the VP usefulness in medical education when issues of development and implementation of VPs are considered. Medical educators are usually encountered with a plethora of educational challenges when asked to transform curricula, or develop PBL cases and courses, or promote independent and active learning. As new technology becomes more widely available for educational purposes, medical educators often take existing training material and simply transcribe it into the new technological medium [28, 29], without considering the yielding of insights upon effective repurposing and curricular incorporation of these educational resources. Recent studies have made efforts to focus on the successful systematic repurposing effort of VPs and their findings are deemed pivotal in an era where open educational resources are transferred to and shared among learners and educators, of various open repositories [28]. Another perspective highlights the use and repurposing of Massive Open Online Courses (MOOCs) extended with VPs, an option that may foster medical skills such as decision making, according to recent studies’ results that have brought insights into the potential benefits, threats, and challenges of MOOOCs and VPs [30].

Efficient educational strategies including current trends in technology and curricula that improve the quality of the medical education system should be widely developed and thoroughly adopted, according to both medical teacher and student perceptions. This will be a very rewarding experience both for those who deliver these educational courses, as well as, for the recipients of such training. Effective teaching using VPs would be beneficial to be set and formally embedded in the medical curriculum. At the same time, a number of remaining issues regarding VPs design, authenticity and implementation need to be fulfilled, in order to reach the potential educational goals of such applications. That way, VPs’ integration in the medical schools, along the lines of innovation and continuous quality improvement and extensive use of analytics [31], are likely to further the medical school as a learning organization with the attendant benefits.

## Limitations

A part of this study included qualitative description. Qualitative description is often criticized for lacking rigour. For this reason, a number of strategies were employed to enhance trustworthiness. Moreover, sampling from a heterogeneous pool of participants (medical teachers of various specialties and medical students of various ages and years of studies) increases the risk for selection bias and makes the comparison of findings difficult.

## Conclusions

We present the results of a combined evaluation of transforming the medical curriculum with VPs’ adoption from both medical teachers’ and students’ perspective. In the face of compelling evidence [13, 32, 33], like those arising from the current study, VPs are effective for teaching and assessment.

The results of this study document high acceptance of VPs by both medical teachers and students, since it is evident that they perceive VPs as an excellent learning tool in undergraduate and postgraduate curriculum. The content of these VP cases has already been repurposed and shared in different educational contexts aiming at a wider adoption of VPs’ integration in medical education [34].

At the same time, important questions that remain on the optimal design, use and integration of VPs should be addressed in order to adequately inform future development. Potential variations in VP design are practically limitless and more research is needed to inform instructional design and curricular integration, so as VPs to be effectively adapted to the needs of potential users and instructors [35]. Future efforts may focus on defining new strategies to integrate traditional types of teaching with the use of VPs in both pre-clinical and clinical curricula. Research should focus on determining ideal topics and learning outcomes in VPs in medical education, identifying optimal exposure to these unique learning content items and addressing the limitations at the same time. Research should also infer if the resulting changes and techniques indeed improve the efficacy of students’ performance, in addition to determining whether VPs in education and assessment have a role to play in mitigating the real clinical skills needed for future doctors.

During the last few years, VPs have become more widely available and easier to create, driven both by student and teacher interest and by recognition of their pedagogic value [36]. Under this perspective, further VP implementations at several medical schools in different universities should be planned to have their role in the curriculum defined and gather useful insights on the factors that make various types of VP curriculum implementations successful. The pace of technological development [37] and the drive to incorporate VP simulations into the curriculum threatens to out-strip our understanding of how they can be used most effectively. If we are to avoid this, we must proceed on a firm basis of an educationally sound designed [38] rigorous evaluation of learning cost-effectiveness, and, above all, provision of adequate training for teaching staff, so as to provide the potential for valid, cost-effective teaching and assessment of clinical skills of medical students. To this extent, this piece of work provides useful and unprecedented insights towards the success of such exercises by academic institutions [39, 40], since it provides a doubled-sided evidence (from both student and teacher perspectives) obtained from a piece-wise but pragmatic application of VP adoption within a medical curriculum. More insights from such exercises are expected in future years as institutions shift emphasis from traditional teaching to problem based learning encounters [41, 42].

## Data Availability

The datasets used during the current study supporting the conclusions of the article are included within the article. The raw data of individual participant will not be shared due to confidentiality agreements approved by the Bioethics Committee.

## Abbreviations

AUTH: Aristotle University of Thessaloniki
eViP: electronic Virtual Patients
IT: Information Technology
KMO: Kaiser-Meyer-Olkin
MOOCs: Massive Open Online Courses
PBL: Problem Based Learning
SPSS: Statistical Package for the Social Sciences
VP: Virtual Patient
